# Waiting for test results before isolating patients with *Clostridium difficile* disease may be associated with increased transmission

**DOI:** 10.1186/2047-2994-4-S1-P26

**Published:** 2015-06-16

**Authors:** M Kiernan, A Chalmers, G Griffiths

**Affiliations:** 1 Richard Wells Research Centre, University of West London, UK; 2 Infection Prevention and Control, Southport and Ormskirk Hospital NHS Trust, Southport, UK

## Introduction

Clostridium difficile is an infection that is associated with environmental contamination. Rapid isolation is recommended to prevent transmission. UK Guidelines state that patients with suspected infection should be isolated within two hours of the onset of symptoms [1], however they also state that the diarrhoea should not be clearly attributable to another cause. This can cause confusion and in busy clinical settings diarrhoea can be attributed to the administration of laxatives and other causes, meaning that patients may not be isolated until after a positive test result is received.

## Objectives

To examine the effect of failure to isolate patients with symptoms of diarrhoea until after a positive test result.

## Methods

Prospective audit of new cases of C. difficile detected after admission by a trained member of the Infection Prevention and Control Team to determine whether the patient from whom a specimen was submitted isolated in a single room facility before a positive test result was notified to the ward.

## Results

There was some evidence of a correlation between higher numbers of C. difficile cases and failure to isolate prior to a positive result for C. diffciile (R= -0.35). Wards that isolated the majority of cases had low numbers of cases.

## Conclusion

Failure to isolate patients with symptoms of diarrhoea could be responsible for increased opportunities for transmission through environmental contamination and increased risk of hand contamination [2]. There are limitations to this work in that the wards are spread across a General Hospital with possibly differing risks of C. difficile disease. No attempt has been made to control for age or other patient-specific risks or antimicrobial use. Further large-scale studies could be undertaken to determine the level of risk presented by non-isolation of patients with potentially infectious diarrhoea until results have been obttained.

## Disclosure of interest

None declared.
